# Evaluation of DNA extraction methods and direct PCR in metabarcoding of mock and marine bacterial communities

**DOI:** 10.3389/fmicb.2023.1151907

**Published:** 2023-04-17

**Authors:** Iva Stojan, Željka Trumbić, Ivana Lepen Pleić, Danijela Šantić

**Affiliations:** ^1^Laboratory of Microbiology, Institute of Oceanography and Fisheries, Split, Croatia; ^2^Doctoral Study of Biophysics, Faculty of Science, University of Split, Split, Croatia; ^3^University Department of Marine Studies, University of Split, Split, Croatia; ^4^Laboratory for Aquaculture, Institute of Oceanography and Fisheries, Split, Croatia

**Keywords:** DNA extraction, mock community, marine bacteria, 16S rRNA, direct PCR, DNA metabarcoding, compositional data analysis

## Abstract

Recent advances in new molecular biology methods and next-generation sequencing (NGS) technologies have revolutionized metabarcoding studies investigating complex microbial communities from various environments. The inevitable first step in sample preparation is DNA extraction which introduces its own set of biases and considerations. In this study, we assessed the influence of five DNA extraction methods [B1: phenol/chloroform/isoamyl extraction, B2 and B3: isopropanol and ethanol precipitations, respectively—both modifications of B1, K1: DNeasy PowerWater Kit (QIAGEN), K2: modified DNeasy PowerWater Kit (QIAGEN) and direct PCR approach (P) that completely circumvents this step on community composition and DNA yield of mock and marine sample communities from the Adriatic Sea]. B1–B3 methods generally produced higher DNA yields and more similar microbial communities, but with higher interindividual variability. Each method demonstrated significant differences in a specific community structure, where rare taxa seem to play a crucial role. There was not one superior method closest to the theoretically expected mock community composition, they all demonstrated skewed ratios, but in a similar way which might be attributed to other factors, such as primer bias or 16S rRNA gene count for specific taxa. Direct PCR represents an interesting approach when high throughput in sample processing is required. We emphasize the importance of making a cautious decision about the choice of the extraction method or direct PCR approach, but even more importantly its consistent application throughout the study.

## 1. Introduction

The continuous and rapid development of new molecular biology methods with now routinely applied next-generation sequencing (NGS) technologies has revolutionized studies investigating the composition and ecology of complex microbial communities originating from various environments, such as marine, freshwater, soil, or various organismal/mucosal microbiomes. Within these scopes, 16S rRNA gene metabarcoding, which grew directly from advantages provided by NGS, has become most widespread method used. Although shotgun metagenomics is increasingly gaining popularity and allows for investigation of functional roles of bacterial communities (Durazzi et al., 2021), due to increased costs and bioinformatic requirements, it still does not fully replace 16S rRNA metabarcoding, especially if taxonomic assignment is the main purpose of large studies. In the absence of current consensus and standardization in molecular biology workflows and data analysis pipelines for 16S rRNA metabarcoding, an in-depth understanding and appreciation of all bias sources contributing to 16S rRNA analyses is required (Knight et al., 2018). First and foremost, DNA extraction methods inevitably introduce specific biases, and together with other contributing factors, result in a poor precision of estimates of relative abundances of a particular taxon (Pollock et al., 2018).

Although many studies have compared frequently used DNA extraction methodologies in terms of bacterial community composition and biodiversity estimates in metabarcoding-based research from various ecosystems (Henderson et al., 2013; Wesolowska-Andersen et al., 2014; Deiner et al., 2015; Walden et al., 2017; Hermans et al., 2018; Liu et al., 2019; Mateus-Barros et al., 2019; Ma et al., 2020), fewer have evaluated them on marine environmental samples (Djurhuus et al., 2017) simultaneously with mock communities (Muñoz-Colmenero et al., 2021). The sequencing of mock communities as “controls” with defined composition together with environmental samples is critical for estimating error rates of NGS runs and necessary for the validation of every novel method proposed (Caporaso et al., 2010; Pollock et al., 2018; Yeh et al., 2018). Most commonly employed DNA extraction methods include various benchmarked kits that usually provide high-quality results but low DNA yield, and/or standard phenol-chloroform-based extraction procedures that utilize hazardous chemicals, but produce high-quality high yield DNA (Renshaw et al., 2015; Muñoz-Colmenero et al., 2021). Experiences from various fields suggest that these procedures may be adjusted to avoid the utilization of hazardous components without compromising high-quality results, such as DNA protein-salting out methods (Aljanabi and Martinez, 1997). Protein salting out protocol has been used in an eukaryotic oriented metabarcoding study (Muñoz-Colmenero et al., 2021), however, it was not directly compared to conventional phenol-chloroform based protocol. These and similar protocols might serve as an alternative to commercially available kits as phenol-free and cost-effective methods, especially with recent concerns about kit residential bacterial contamination (Salter et al., 2014).

With the constant improvements in the biotechnology industry, it is also possible to skip DNA extraction step and proceed with direct PCR amplification of the sample matrix. Even though the direct PCR approach has been recognized for many years as potentially one of the most useful molecular techniques that have been applied to a wide range of biological studies and biomedical diagnoses, its application in environmental microbiology, to our knowledge, remains somewhat neglected (Ben-Amar et al., 2017; Vinayaka et al., 2019; Tao et al., 2022). Since direct PCR amplification is carried out directly on samples, this powerful, straightforward, and time-efficient method which utilizes high-performance DNA polymerases is particularly useful when handling a large number of samples, as frequently is the case with laborious metabarcoding-based research (Ben-Amar et al., 2017).

In this study, we provide simultaneous comparison of five DNA extraction protocols (three biochemically- and two kit-based methods) and one direct PCR approach on mock and marine bacterial communities using 16S rRNA metabarcoding with compositional data analysis. Environmental samples were collected from two locations in the Eastern Central Adriatic Sea at two sampling time points (2020 and 2022). To our knowledge, this is the first application of direct PCR amplification of the V4–V5 hypervariable region of the 16S rRNA marker gene on seawater samples. Between biochemically based DNA extraction methods, we propose two modifications of phenol-based protocol that are less time-consuming, cost-effective and significantly less hazardous.

## 2. Materials and methods

### 2.1. Preparation of mock communities and marine environmental samples collection

Nine prokaryotes cell suspensions originating from marine environments were purchased as pure cultures from The Roscoff Culture Collection (France), including the *Synechococcus* sp. (RCC48), *Roseovarius tolerans* (RCC1914), *Flavobacteriaceae* (RCC5730), *Bacillus* sp. (RCC6828), *Pseudoalteromonas undina* (RCC4305), *Microbacterium* sp. (RCC4176), *Vibrio* sp. (RCC4144), *Erythrobacter* sp. (RCC1876), and *Glaciecola* sp. (RCC4342). *Synechococcus* sp., *Glaciecola* sp., *Vibrio* sp., *Microbacterium* sp., and *Pseudoalteromonas undina* originated from pure but non-axenic cultures, while other bacterial cultures were stated to be pure and axenic. These particular bacteria were chosen to create an even mock community (11.11% of each bacterial species) since they represented five different prokaryotic orders isolated mainly from the Mediterranean Sea, therefore could be potentially found in the Adriatic as well. The cell concentration in each culture was measured with a CytoFLEX flow cytometer (The Beckman Coulter, USA) equipped with laser emission at 488 nm. Nine different bacterial isolates were thoroughly resuspended in 1 mL of sterile artificial seawater (37 g NaCl dissolved in 1L Milli-Q). Resuspended bacteria were then diluted to targeted concentrations and all isolates were pooled in 1 L of artificial seawater in a borosilicate glass bottle (SCHOTT, The United Kingdom) to obtain a final mixture composed of the same number of each of the nine species with the total cell concentration of 3.053 × 10^4^ cells mL^–1^. This total count is comparable to the smallest number of bacteria per mL recorded in the Southern Adriatic Sea (Šantić et al., 2021).

Fifty mL aliquots represented technical replicates and were vacuum-filtered through 0.22-μm polyethersulfone membrane filters (PES, 47 mm diameter, FiltraTECH, France) which were immediately stored after filtration at −80°C until DNA extraction (within 2 weeks).

Marine environmental samples were collected from two sites in the eastern central Adriatic Sea in October 2020 in Kaštela Bay (43.52 N; 16.37 E, ∼300 m offshore) and in February 2022 at Strožanac beach (43.50 N; 16.53 E), Croatia, near the city of Split. Approximately 32 L altogether of the surface layer from these two sites (upper ∼30 cm) were collected in bottles and prefiltered through a 20-μm plankton net. Within 1 h after sampling, aliquots of 1 L were vacuum-filtered through 0.22-μm polyethersulfone membrane filters (PES, 47 mm diameter, FiltraTECH, France). To ensure better methods’ inter-comparability, 1 L was chosen as a fixed filtration volume for all samples (Boström et al., 2004). After filtration of seawater, filters were immediately frozen at −80°C until further DNA extraction (within 2 weeks). One liter of Milli-Q water represented a negative filtration control (five filters in total, one PES filter per extraction method).

### 2.2. DNA extraction methods, quality control, and PCR amplifications

Five DNA extraction protocols and direct PCR (Table 1) were tested on a total of 26 mock and 31 environmental seawater samples from two different locations. All filtration technical replicates (minimum two for 2020 marine samples, maximum six for mock) within each method came from the same collected seawater or mock community mixture. Negative controls (extraction blanks) included empty 0.22-μm polyethersulfone membrane filters (PES, FiltraTECH, France) run through the filtration process and all extraction methods tested. Elution/dissolution volume was fixed at 35 μL for all extraction methods to allow standardization of DNA yield/concentration, used as a proxy for estimating each method’s efficiency. Filtration, all DNA extractions, and PCR amplifications were performed in the same laboratory by the same person.

**TABLE 1:** Specifications of conventional and modified DNA extraction methods and direct PCR evaluated in this study.

Method	Abbreviation	DNA extraction	Lysis method	DNA Polymerase in PCR amplification	Advantages	Disadvantages
Phenol/chloroform/isoamyl	B1	Yes	Heat, biochemical	Q5^®^ high-fidelity (NEB)	Low cost per sample, satisfactory DNA yields and qualities	Severely hazardous, lengthy protocol (2 days)
Isopropanol precipitation	B2	Yes	Heat, biochemical	Q5^®^ high-fidelity (NEB)	Not toxic, low cost per sample, satisfactory DNA yields and qualities	Lengthy protocol (2 days)
Ethanol precipitation	B3	Yes	Heat, biochemical	Q5^®^ high-fidelity (NEB)	Not toxic, low cost per sample, satisfactory DNA yields and qualities	Lengthy protocol (2 days)
DNeasy PowerWater Kit (Qiagen)	K1	Yes	Mechanical, chemical	Q5^®^ high-fidelity (NEB)	Rapid and straightforward protocol	High cost per sample, low DNA yield, a lot of environmental bacteria not detected
Modified DNeasy PowerWater Kit (Qiagen)	K2	Yes	Mechanical, chemical	Q5^®^ high-fidelity (NEB)	Rapid and straightforward protocol	High cost per sample, low DNA yield, a lot of environmental bacteria not detected
Platinum direct PCR universal master mix (Invitrogen, Thermo Fisher Scientific)	P	No	Heat, chemical	Invitrogen Platinum II Taq Hot-Start	Rapid, robust and optimized protocol, low cost per sample, DNA extraction circumvented	No DNA available for other purposes (qPCR, metagenomics)

#### 2.2.1. Phenol/chloroform/isoamyl extraction (method one–B1)

The widely used conventional phenol/chloroform/isoamyl extraction was the first method of choice (Renshaw et al., 2015; Djurhuus et al., 2017; McKiernan and Danielson, 2017). Briefly, PES filters were cut in half with a sterilized scalpel into smaller pieces and 750 μL of TEN lysis buffer (1 M Tris–HCl, 1 M NaCl, 500 mM EDTA) were added to each tube and incubated for 1 h at room temperature. Next, 25 μL of lysozyme (1 mg/mL final conc.) were added and incubation at 37°C for 1.5 h followed. Eight μL of proteinase K (0.2 mg/mL final conc.) and 40 μL 20% SDS were added and the reaction mixture was incubated for 1 h at 65°C in the heating block, followed by heating at 95°C for 10 min. Following this, 750 μL of premixed phenol/chloroform/isoamyl alcohol (25:24:1, BioUltra, Sigma Aldrich-Merck, Germany) were added, samples were vigorously shaken and centrifuged for 10 min at 18000 RCF. The upper layer was transferred to a new tube (∼700 μL), mixed with chloroform/isoamyl alcohol (24:1), and centrifuged for 10 min at 18000 RCF. The upper aqueous phase containing DNA (∼600 μL) was transferred to a new tube, mixed with 1400 μL ice-cold absolute ethanol, vortexed for 5 s, and incubated overnight at −20°C. The next day, samples were centrifuged for 30 min at 20000 RCF and 4°C and washed twice with 500 μL cold 70% ethanol followed by centrifugation for 10 min at 20000 RCF and 4°C. Ethanol was removed carefully and pellets were dried at 37°C for 5 min. Dry pellets were resuspended in 35 μL of 1 × TE (10 mM Tris, 1 mM EDTA, pH ∼8.0) buffer.

Total extracted DNA was quantified and qualities (A260/A280 and A260/A230 absorbance ratios) were measured with the DS-11 Spectrophotometer (Denovix, USA) according to the manufacturer’s instructions. Negative extraction controls showed DNA concentrations below the limit of detection. DNA was considered pure for subsequent analyses if A260/A280 nm ratio was between 1.8 and 2.0 (Hermans et al., 2018). For extracted DNA visualization, routine 1% agarose gel electrophoresis of a subset of samples was performed.

DNA isolated from mock communities and environmental samples were amplified using 515F-Y (5′-GTGYCAGCMGCCGCG GTAA-3′) and 926R (5′-CCGYCAATTYMTTTRAGTTT-3′) primer pair targeting V4-V5 hypervariable regions of the 16S rRNA gene (Parada et al., 2016). Each extraction replicate was amplified in triplicates where 25 μL reaction mixture for each sample contained 12.5 μL Q5^®^ High-Fidelity 2X Master Mix (New England Biolabs, USA), 1.25 μL of each primer at final concentration 0.5 μM, 1 μL of DNA template (conc. 1 ng/μL) and 9 μL of sterile, nuclease-free water. Cycling conditions were as follows: initial denaturation at 98°C for 30 s, followed by 25 cycles of amplification at 98°C for 7 s, 60°C for 30 s and 72°C for 30 s, with 2 min of final extension at 72°C (T100 thermal cycler, Biorad, USA).

#### 2.2.2. Modified phenol/chloroform/isoamyl extraction (isopropanol and ethanol precipitation protocols–methods two and three, B2 and B3, respectively)

Similar to the previously described B1 protocol, modified, shorter, and less hazardous versions of it without phenol/chloroform/isoamyl alcohol and chloroform/isoamyl alcohol were also evaluated (Harding et al., 2011). All the steps of the protocol before the phenol/chloroform/isoamyl addition step were the same as described in method B1. After incubation at 95°C, samples were centrifuged for 20 min at 18000 RCF. The supernatant (∼700 μL) was carefully transferred to a new tube avoiding the cell debris. Half of the samples were mixed with one volume of isopropanol (B2), while the other half with two volumes of ice-cold absolute ethanol (B3). After overnight incubation at −20°C, the DNA was precipitated, washed, dried, and resuspended identically as in B1. Detailed protocol for ethanol and isopropanol precipitation is given in Supplementary Data 1. Quality control and PCR amplifications were performed as described for B1.

#### 2.2.3. DNeasy PowerWater Kit (QIAGEN) (method four–K1)

DNA extractions with DNeasy PowerWater Kit (QIAGEN, Netherlands) were performed according to the manufacturer’s instructions with slight modifications to improve DNA yield: 15 min vortex instead of 5 min vortex at maximum speed (Step 7 in Quick-Start Protocol) with bead-beating tubes positioned horizontally and final elution performed with 35 μL of EB solution (Step 21 in Quick-Start Protocol). Important to note, due to the discontinuation of the garnet beads accelerated by a supply shortage in 2021, QIAGEN replaced them with ceramic ones of various diameters (PowerBead Pro technology) and stated that performance was comparable to or even better than that of garnet ones. Samples collected in 2020 were processed with the 2020 batch (LOT 166020493) which utilized visibly larger garnet beads while samples collected in 2022, as well as mock community samples, were processed using the 2022 batch (LOT 169044629) that came with a mix of various sizes of ceramic beads. Quality control and PCR amplifications were performed as described for B1.

#### 2.2.4. Modified DNeasy PowerWater Kit (QIAGEN) protocol (method five–K2)

In an attempt to additionally improve the DNA yield after extraction with K1, the modified protocol was tested with the following changes: MagNA Lyser Green Beads (Roche, Switzerland, 1.4-mm ceramic beads) were introduced instead of manufacturer’s bead tubes. PES filters were cut in half with a sterilized scalpel and put in 1.5 mL tubes filled with ceramic beads followed by rigorous homogenization with MagNALyser Instrument (Roche, Switzerland), twice for 20 s at 9000 RCF. After homogenization, tubes were centrifuged for 1 min at 5600 RCF. The supernatant was transferred to a clean 2 mL collection tube and the protocol proceeded as per manufacturer’s instructions (from Step 8 in Quick-Start Protocol). In this case, elution was performed with 35 μL of EB solution as in K1. Quality control and PCR amplifications were performed as described for B1.

#### 2.2.5. Direct PCR using Platinum Universal Master Mix (method six–P)

For direct amplification of the sample matrix (PES filter), Platinum Direct PCR Universal Master Mix (Invitrogen, Thermo Fisher Scientific, USA) was used according to the manufacturer’s instructions. A short lysis protocol suggested by the manufacturer was performed for each filter as follows: 3 μL of Proteinase K were added to 100 μL Lysis Buffer (provided by the manufacturer), mixed briefly by vortexing, and spun down. A miniature piece of PES filter (approximately 1/10 of the filter, cut from the middle to the edges) was entirely emerged into the lysis solution, incubated at room temperature for 5 min, and then incubated at 98°C for 1 min. The choice of using a small piece of filter was made according to manufacturer guidelines for size of input material. Alternatively, 1 μL of seawater as a template could be used, however, we believe this would have introduced even a greater bias in respect to other methods tested. A parallel sample was processed using a clean filter to serve as a negative control for PCR amplification. The lysate was briefly centrifuged and 1 μL of lysate supernatant served as a template to prepare the PCR reaction mix for the 16S rRNA gene amplification targeting the V4–V5 hypervariable regions with 515F-Y (5′-GTGYCAGCMGCCGCGGTAA-3′) and 926R (5′-CCGYCAATTYMTTTRAGTTT-3′) primer pair (Parada et al., 2016). All PCR reactions were performed in triplicates. For each sample, the 20 μL reaction mixture consisted of 10 μL 2X Platinum Direct PCR Universal MasterMix, 0.4 μL of each primer at the final concentration of 0.2 μM, 1 μL of lysate supernatant and 8.2 μL of sterile, nuclease-free water. Cycling conditions were followed according to the manufacturer’s instructions: 94°C for 2 min, followed by 35 cycles of 94°C for 15 s, 60°C for 15 s and 68°C for 20 s (T100 thermal cycler, Biorad, USA).

### 2.3. PCR product purification, library preparation, and sequencing

Agarose gel (1.5%, stained with GelRed, Sigma Aldrich, USA) electrophoresis in 1 × TAE buffer was performed to visualize PCR products and negative controls. All extraction blanks and non-template controls were negative. Triplicates from PCR amplification were pooled and purified using the GeneJET PCR Purification Kit (Thermo Scientific, USA) according to the manufacturer’s instructions. To quantify purified PCR product concentrations, DS-11 Spectrophotometer (Denovix, USA) was used according to the manufacturer’s instructions. Library preparation and pair-end amplicon sequencing (2 × 250 bp) on the Illumina MiSeq of V4-V5 16S rRNA regions were performed by the Genomics Core Facility of the Universitat Pompeu Fabra (Barcelona, Spain), according to Illumina 16S Metagenomic Sequencing Library Preparation guidelines (15044223 Rev. B).^1^ Each extraction replicate was sequenced as an individual sample.

### 2.4. Bioinformatics, statistical analysis, and data visualization

To statistically compare differences in DNA yield between different DNA extraction methods, the non-parametric Kruskal–Wallis test followed by *post hoc* Dunn’s multiple comparisons test was performed in R (v4.2.2).

Concerning current advances in microbiome data analysis, despite rarefying still being a widely popular normalization technique in ecological studies, we implemented compositional approach in this research (Fernandes et al., 2014; McMurdie and Holmes, 2014; Gloor et al., 2017). Compositional data analyses of NGS results are currently becoming more widespread with an emphasis that microbiome datasets can and should be treated as compositional (Tsilimigras and Fodor, 2016; Gloor et al., 2017; Sisk-Hackworth and Kelley, 2020; Harrison et al., 2021).

A total of 2029701 input reads from two Illumina runs were used in bioinformatic analyses and initial counts per sample are given in Supplementary Table 1. The quality of raw paired-end reads was evaluated using FastQC v0.11.9. Following the removal of MiSeq adapters and barcodes, primers were trimmed using cutadapt v4.1 (Martin, 2011). Subsequent data processing was carried out in R/Bioconductor with package dada2 v1.16.0 (Callahan et al., 2016). Mock community and environmental samples were analyzed separately, due to the specific parametric error model in the learnErrors function which dada2 implements to distinguish sequencing error rates between distinct sample types (Caporaso et al., 2010). Briefly, filterAndTrim function [truncLen = c (230,225), maxN = 0 (no N allowed), maxEE = c (2, 2), truncQ = 2, rm.phix = TRUE] filtered out low quality sequences and tails. Error learning and sample inference followed by merging of paired-end reads resulted in the amplicon sequence variant (ASV) table. Default removeBimeraDenovo function with the “pooled” method was used to remove chimeras, which contributed from 2.22 to 2.81% of the merged sequence reads for mock and environmental samples, respectively (Supplementary Table 1). The assignTaxonomy function implementing the naive Bayesian classifier method was used to assign the taxonomy using Silva v138.1 database, updated on March 10, 2021 (Quast et al., 2013). After creating a phyloseq object with the phyloseq R package v1.32.0, ASVs classified as “chloroplast” or “mitochondria” were discarded from further analyses (McMurdie and Holmes, 2013). Rare ASVs not represented with at least five reads in three samples (smallest experimental group per extraction method) were removed from analyses and ASVs were agglomerated to the genus level. Mock dataset was additionally filtered specifically to mock taxa to obtain the core community originally added, followed by agglomeration of ASVs to the family level. This served to better explore the relationships between mock taxa only. Several mock ASVs classified as order Bacillales in Silva database, were manually annotated using blastn with NCBI nr as *Bacillus* sp. (query coverage 100%, *E*-value 0.0 and percent identity 100.0), congruent with taxa used for mock community.

Addressing the compositional nature of the microbiome count data, centered log-ratio (clr) transformation was performed for mock and environmental datasets separately, by taking the log-ratio of each taxa value in the sample divided by the geometric mean of all the counts for that sample (Aitchison, 1982; Gloor et al., 2017; Sisk-Hackworth and Kelley, 2020). The transformation was performed as implemented in microbiome v1.10.0 R package *via* transform function that introduces pseudo-counts of the minimum relative abundance divided by two to substitute zero abundance entries in the ASV table before taking the log-ratio (Lahti et al., 2017). To test the significance of the effect of the DNA extraction method on bacterial community composition, permutational multivariate analysis of variance (PERMANOVA) based on Aitchison distances was performed in PRIMER7 (Clarke and Gorley, 2015). PERMANOVA was performed with the “method” as a fixed factor [9999 permutations, sums of squares type: Type III (partial), permutation method: Unrestricted permutation of raw data] (Anderson, 2017). Contrasts to compare different methods were designed according to the main experimental questions, i.e., comparison of direct PCR, kit based and biochemically based DNA extraction methods.

Variance-based compositional principal component (PCA) biplots on Aitchison distances were generated based on clr-transformed values for a particular taxonomic rank (genus or family) with zero replacement using pseudo-counts with the R package microViz v0.10 (Aitchison et al., 2000; Gloor et al., 2017; Barnett et al., 2021). Heatmap presentation of taxa compositions was generated using heatmap.2 function of gplots v3.1.3 package based on Aitchison distances and Ward. D2 dendrogram agglomeration method (Warnes et al., 2022). R package ggplot2 v3.3.5 and patchwork v1.1.2 were used throughout the manuscript for all visualizations (Wickham, 2016). To estimate the number of shared ASVs between methods, Venn diagrams were generated using the ps_venn function from the R package MicEco v0.9.18 on both unfiltered and filtered environmental datasets (Pedersen, 2022; Russel, 2022).

### 2.5. Data availability

Sequence data are available in the NCBI database as a part of BioProject PRJNA912619 under accession numbers SAMN32769568-SAMN32769624.

## 3. Results

### 3.1. Evaluation of DNA extraction methods for mock community

Five DNA extraction protocols (B1, B2, B3, K1, K2) and direct PCR amplification (P) were tested on mock community samples consisting of the same amounts of nine marine bacterial species originating from both axenic and non-axenic pure cultures: *Synechococcus* sp., *Roseovarius tolerans*, *Flavobacteriaceae*, *Bacillus* sp., *Pseudoalteromonas undina*, *Microbacterium* sp., *Vibrio* sp., *Erythrobacter* sp., and *Glaciecola* sp.

#### 3.1.1. DNA yields and direct PCR

**FIGURE 1:**
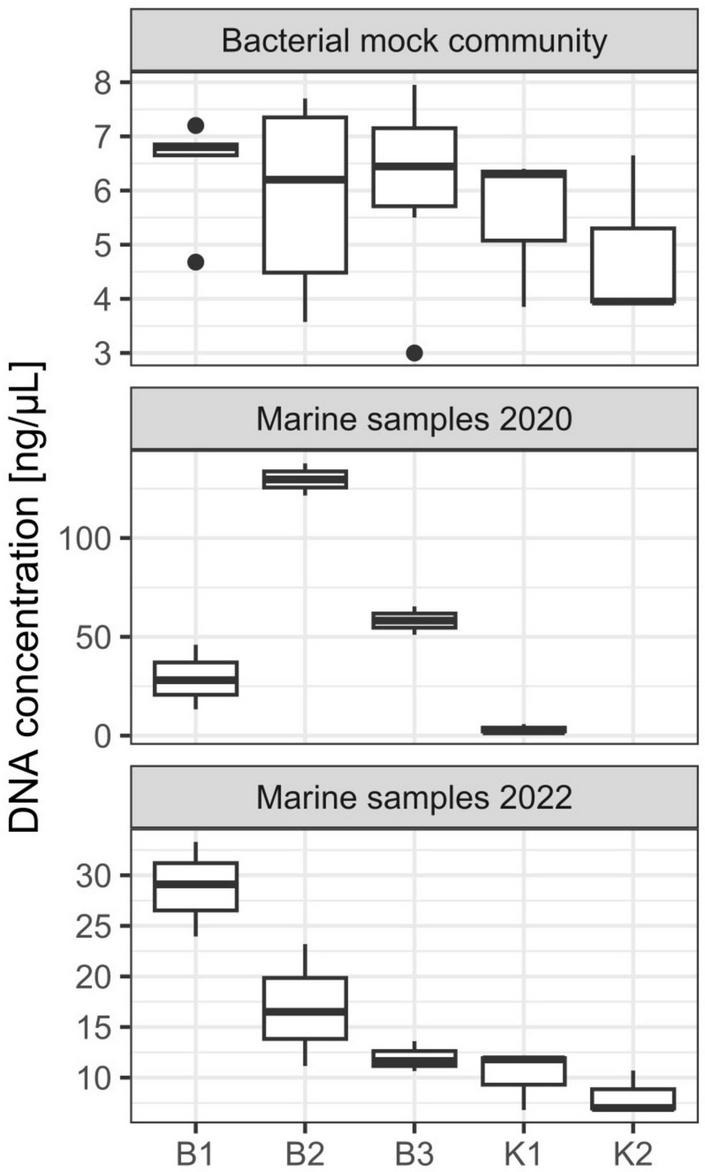
DNA yield (ng/μL) from DNA extractions performed on mock community and marine samples from the eastern central Adriatic Sea in 2020 and 2022 with different extraction methods [B1: conventional phenol/chloroform/isoamyl extraction, B2 and B3: isopropanol and ethanol precipitations, respectively, K1: DNeasy PowerWater Kit (QIAGEN), K2: modified DNeasy PowerWater Kit (QIAGEN), P: direct PCR, Platinum Universal Master Mix (Invitrogen, Thermo Fisher Scientific)]. The lower and upper hinges correspond to the 25th and 75th percentiles. The upper whisker extends from the hinge to the largest value no further than 1.5 × IQR from the hinge (where IQR is the inter-quartile range or distance between the first and third quartiles). The lower whisker extends from the hinge to the smallest value at most 1.5 × IQR of the hinge. Data beyond the end of the whiskers are called “outlying” points and are plotted individually.

Three DNA extraction protocols (B1, B2, and B3) evaluated in this study are based on the biochemical lysis of cells with lysozyme, proteinase K, and sodium dodecyl sulfate, whilst two (K1 and K2) apply bead-beating (mechanical) disruption of cells in combination with a kit specific lysis solution. All extraction methods yielded DNA that could be quantified spectrophotometrically and all samples had an acceptable DNA purity for successful PCR amplifications. Agarose gel electrophoresis of extracted genomic DNA revealed high-molecular-weight genomic DNA yielded with B1, B2, and B3 protocols, while smearing was observed for K1 and K2 (data not shown). No significant differences in DNA yield were observed between the five different methods (Kruskal-Wallis test, chi-squared = 2.9889, df = 4, *p*-value = 0.5597). Generally, the lowest yields were observed for methods K1 and K2, based on DNeasy PowerWater Kit with the tendency to increase for B1, B2, and B3 methods (Figure 1 and Supplementary Table 2). Based on a range of observed values, B1 showed the highest reproducibility regarding DNA yield obtained whilst the highest variation among samples was observed for B2 (Figure 1). Purities of DNA extracts, A260/A280 and A260/A230 absorbance ratios were generally higher (>1.8) and lower (<2.0), respectively, than is usually accepted for PCR amplification (Supplementary Table 2), nonetheless, all samples resulted in successful amplification. Regarding direct PCR (P), even though as little as 1 μL of lysis buffer with DNA of interest was used as a template for 16S rRNA gene amplification, all reactions in triplicates for each technical replicate were successful.

#### 3.1.2. 16S rRNA gene amplicon sequencing results

The maximum mean number of reads per sample produced by MiSeq Illumina sequencing with the highest variability was observed for P (177 142.33, SD 225 884.66), followed by K1 and K2 (132 469, SD 95 090.78; 114 855.67, SD 29 117.05, respectively). The mean number of reads per sample for B2 and B3 was 43 789 (SD 37 704.48) and 82 594.50 (SD 104 216.49) respectively. The lowest overall values of reads per sample were observed for B1 (mean 5063.80, SD 4599), where one sample was discarded from the following analyses since it had an unacceptably low number of input reads (348) (Supplementary Table 1).

#### 3.1.3. Mock community composition

**FIGURE 2:**
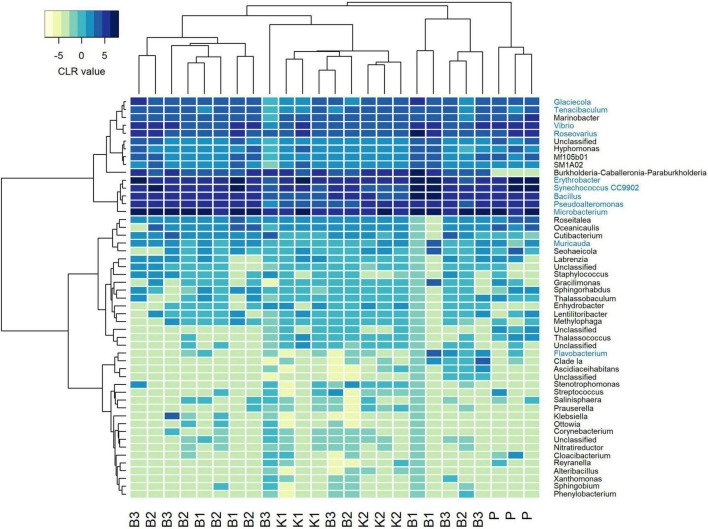
Heatmap depicting Aitchison distances of clr-transformed filtered mock data agglomerated at genus level with Ward. D2 dendrogram agglomeration method. Mock constituents are colored blue. B1: conventional phenol/chloroform/isoamyl extraction, B2 and B3: isopropanol and ethanol precipitation, respectively, K1: DNeasy PowerWater Kit (QIAGEN), K2: modified DNeasy PowerWater Kit (QIAGEN), P: direct PCR, Platinum Universal Master Mix (Invitrogen, Thermo Fisher Scientific).

**FIGURE 3:**
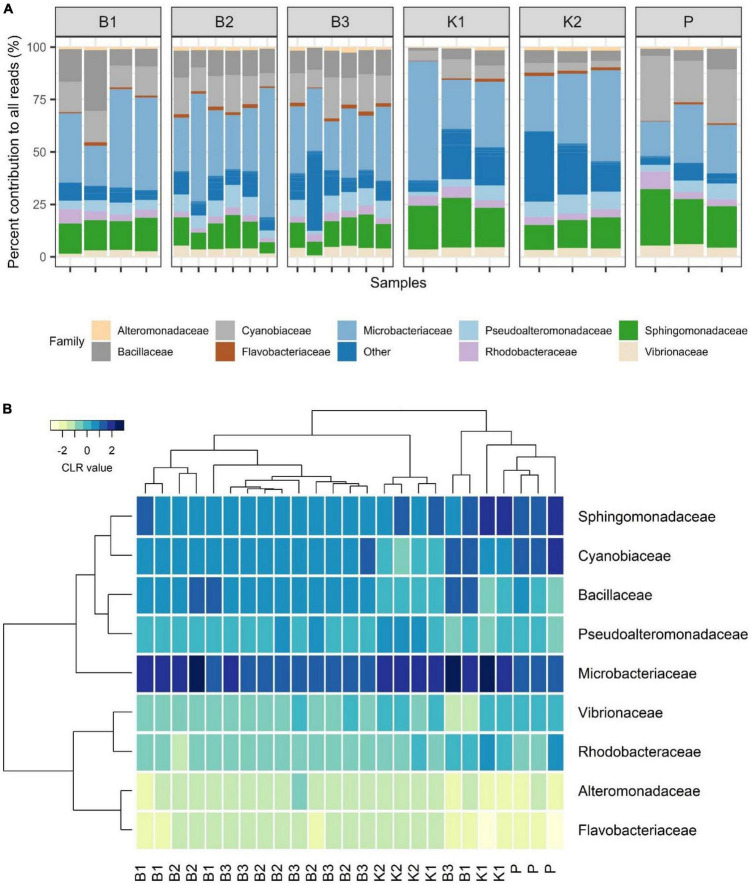
**(A)** Barplot showing relative abundances of all mock constituents at the family level with non-mock taxa shown as“Other.” **(B)** Heatmap depicting Aitchison distances of clr-transformed core mock data agglomerated at family level with Ward. D2 dendrogram agglomeration method. B1: conventional phenol/chloroform/isoamyl extraction, B2 and B3: isopropanol and ethanol precipitation, respectively, K1: DNeasy PowerWater Kit (QIAGEN), K2: modified DNeasy PowerWater Kit (QIAGEN), P: direct PCR, Platinum Universal Master Mix (Invitrogen, Thermo Fisher Scientific).

Regarding mock community composition, all extraction methods successfully detected every artificially added prokaryote at the genus level, however, many other taxa were detected as well (Figure 2). Data processing resulted in a total of 670 ASVs, of which 140 had taxonomic assignments of intended mock taxa. In order to inspect the relationships between core mock taxa on their own and with other non-target ASVs detected across methods, after filtering of rare ASVs, analyses were applied to two sets: (1) one subsetted to taxa included in mock and agglomerated at the family level, referred to as core mock (N of taxa = 9); and (2) complete dataset agglomerated at the genus level (N of taxa = 52). We acknowledge that subsetting data to mock taxa only represents the subcomposition of entire data which might introduce inconsistencies (Gloor et al., 2017), however, they represent the main target taxa which need to be inspected on their own. Agglomeration of data at the family level was necessary to collect mock taxa as a single feature. Even though individual isolates were mixed in an equal theoretical proportion of 11.11%, differences in relative abundances were observed, similarly skewed across all methods (Figure 3A). Relative proportion of other non-target taxa ranged from 4 to 38% per sample, with smallest average contribution recorded for direct PCR (5.9%) and largest for kit based method K2 (24.2%). Relative abundances for mock community constituents and other non-target taxa aggregated at the family level are given in Supplementary Data 2. By large, relative abundances of *Microbacterium* sp. were the highest, dominating the mock community composition across all methods with average relative abundances of 33.13%. *Erythrobacter* and *Synechococcus* were marginally over-represented. *Bacillus* was marginally over-represented in B1 and under-represented across K1, K2, and P. *Vibrio*, *Pseudoalteromonas*, *Roseovarius*, and *Glaciecola* were all under-represented across all methods (Figure 3A). Generally, the most under-represented bacteria across all samples seem to be *Flavobacteriaceae*.

In the compositional overview of core mock taxa alone, similar trends were observed (Figure 3B), with *Microbacterium* sp. (Microbacteriaceae) dominating the community and *Flavobacteriaceae* being least represented. Clustering of samples per DNA extraction method in the heatmap indicated grouping of B1/B2/B3 methods (phenol/isopropanol/ethanol precipitation), however, not in a unique cluster, while K2 (modified PowerWater Kit) clustered as a group with one K1 sample. Direct PCR (P) was placed in a cluster of its own (Figure 3B).

**FIGURE 4:**
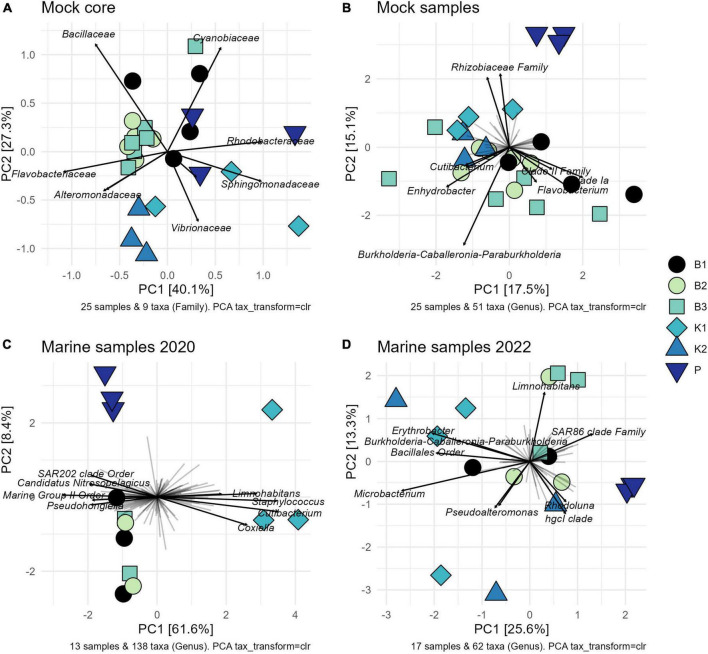
Variance-based compositional principal component (PCA) biplots on Aitchison distances on clr-transformed values with zero replacement using pseudo-counts, showing groupings by extraction method and top taxa by the longest line length: **(A)** core mock taxa agglomerated at family level; **(B)** entire mock community agglomerated at genus level; **(C)** 2020 marine samples agglomerated at the genus level after rare ASVs removal; **(D)** 2022 marine samples agglomerated at the genus level after rare ASVs removal. B1: conventional phenol/chloroform/isoamyl extraction, B2 and B3: isopropanol and ethanol precipitation, respectively, K1: DNeasy PowerWater Kit (QIAGEN), K2: modified DNeasy PowerWater Kit (QIAGEN), P: direct PCR, Platinum Universal Master Mix (Invitrogen, Thermo Fisher Scientific).

Consistently, groupings among methods were not directly evident from the PCA biplot based on Aitchison distances of clr-transformed data for core mock taxa (Figure 4A). K2 samples clustered tightly together, while greater variability of B1 (phenol) samples was observed. B2 and B3 generally grouped together, with the exception of one B3 sample. Results of statistical comparisons of sample compositions between DNA extraction methods using PERMANOVA are outlined in Table 2. At the general level, the differences in core mock community were statistically significant (Pseudo-F = 4.2468, *p* = 0.0001). Taking contaminant taxa into account at the genus level, P (direct PCR) separated to a greater extent from other methods, while K2 maintained their tight positioning and were closely placed to K1 (Figure 4B). B1-B3 biochemically based protocols demonstrated greater variability. As with core mock data, PERMANOVA results indicate there is a significant difference in mock composition between experimental approches (Pseudo-F = 1.9394, *p* = 0.0002). What is observed on both sets is that there is no difference between isopropanol and ethanol precipitation methods (between B2 and B3) and in respect to phenol/chloroform/isoamyl method (B1 vs. B2/B3). In respect to biochemical methods, kit based ones and direct PCR differ significantly. Biplot loadings suggest the main taxon driving separation of B1/B2/B3 is *Bacillus*, and the contribution of contaminant taxa is considerable. For instance, Burkholderia-Caballeronia-Paraburkholderia was not detected in P (direct PCR), driving the separation of P samples from the rest. This might underline the possible advantage of lowered sample manipulation time in the laboratory. Burkholderia-Caballeronia-Paraburkholderia were not introduced through usage of non-axenic cultures as they do not appear in direct PCR, while all mock taxa do. The importance of data filtering is also outlined, however, complete contamination removal by automatic criteria was not possible without the loss of true mock taxa. Raw ASV counts for the mock community library are provided in Supplementary Data 3, including taxonomy assignments and sample data.

**TABLE 2:** Permutational multivariate analysis of variance (PERMANOVA) for comparisons of mock and marine communities between five different DNA extraction methods and direct PCR based on Aitchison distances of clr-transformed data [B1: conventional phenol/chloroform/isoamyl extraction, B2 and B3: isopropanol and ethanol precipitation, respectively, K1: DNeasy PowerWater Kit (QIAGEN), K2: modified DNeasy PowerWater Kit (QIAGEN), P: direct PCR, Platinum Universal Master Mix (Invitrogen, Thermo Fisher Scientific)].

Contrast	Source	df	SS	MS	Pseudo-F	P (perm)	Unique perms
**PERMANOVA table of results (core mock, family level)**							
	Method	5	20.094	4.0188	4.2468	0.0001*	9906
(B2) v (B3)	C1	1	0.43299	0.43299	0.54087	0.6746	462
(B1) v (B2, B3)	C2	1	1.7692	1.7692	2.1648	0.0761	1805
(P) v (B1, B2, B3)	C3	1	6.2517	6.2517	6.6859	0.0023*	968
(K1, K2) v (B1, B2, B3)	C4	1	8.1637	8.1637	7.6503	0.0003*	9298
(K1, K2) v (P)	C5	1	5.8656	5.8656	3.796	0.0597	84
	Res	19	17.98	0.94632			
	Total	24	38.074				
**PERMANOVA table of results (filtered mock, genus level)**							
	Method	5	892.05	178.41	1.9394	0.0002*	9830
(B2) vs. (B3)	C1	1	79.668	79.668	0.73504	0.7629	462
(B1) vs. (B2, B3)	C2	1	168.98	168.98	1.5807	0.0784	1806
(P) vs. (B1, B2, B3)	C3	1	354.91	354.91	3.3088	0.0008*	966
(K1, K2) vs. (B1, B2, B3)	C4	1	231.75	231.75	2.4391	0.0025*	9225
(K1, K2) vs. (P)	C5	1	300.04	300.04	5.3484	0.0116*	84
	Res	19	1747.9	91.994			
	Total	24	2639.9				
**PERMANOVA table of results (2020 marine samples, genus level)**							
	Method	4	1763.4	440.84	5.4558	0.0002*	9810
(B1) vs. (B2, B3)	C1	1	92.178	92.178	1.7869	0.0262*	35
(P) vs. (B1, B2, B3)	C2	1	183.91	183.91	3.1913	0.0077*	120
(K1) vs. (B1, B2, B3)	C3	1	1270.8	1270.8	15.355	0.0073*	120
	Res	8	646.42	80.802			
	Total	12	2409.8				
**PERMANOVA table of results (2022 marine samples, genus level)**							
	Method	5	517.79	103.56	1.6437	0.0048*	9835
(B1) vs. (B2, B3)	C1	1	74.256	74.256	1.2559	0.1442	28
(P) vs. (B1, B2, B3)	C2	1	144.08	144.08	2.8863	0.0055*	165
(K1, K2) vs. (B1, B2, B3)	C3	1	144.09	144.09	2.0261	0.0052*	2911
(K1, K2) vs. (P)	C4	1	236.64	236.64	3.7253	0.0119*	84
	Res	11	693.04	63.004			
	Total	16	1210.8				

*P value < 0.05.

### 3.2. Evaluation of DNA extraction methods for environmental samples

In addition to mock community samples, six different methodologies on a total of 31 marine samples (technical replicates) from two different locations and time points were evaluated to compare bacterial community structure between methods. All methods were always compared within a single sampling time/place.

#### 3.2.1. DNA yields and direct PCR

Concerning marine samples collected in October 2020 in Kaštela Bay, B2 resulted in the highest DNA yield, followed by B3 and B1 (Figure 1). The lowest DNA yield were again observed for K1, however, we must emphasize that these extractions were performed using older batches of DNeasy PowerWater Kit from the 2020 year and that the manufacturer (QIAGEN) replaced the bead-beading technology with, as they stated, a superior one in 2021. Statistically significant differences between extraction methods in terms of DNA yields were detected (Kruskal-Wallis test, chi-squared = 8.4545, df = 3, *p*-value = 0.03749). *Post hoc* Dunn’s test revealed significant differences between isopropanol/ethanol and kit based methods (B2 and K1; B3 and K1 *p* < 0.05). K2 was not evaluated on these samples. A260/A280 and A260/A230 ratios were satisfactory for downstream applications (Supplementary Table 2). Direct PCR amplification in triplicates from PES filters was successful for all environmental samples.

Similarly to mock communities, B1 resulted in highest DNA yield obtained from marine samples collected in February 2022 in Strožanac Beach, followed by B2 and B3 (Figure 1). The lowest yields were observed for K1 and K2. The differences detected were statistically significant (Kruskal-Wallis test, chi-squared = 9.8333, df = 4, *p*-value = 0.04333). B3 protocol showed the highest reproducibility among the methods assessed. *Post hoc* Dunn’s test revealed significant differences between phenol and kit based methods (B1 and K1; B1 and K2, *p* < 0.05). Purities of DNA extracts, A260/A280 and A260/A230 absorbance values were sufficient for successful PCR amplifications (Supplementary Table 2).

#### 3.2.2. 16S rRNA gene amplicon sequencing results

The maximum average coverage with the highest variability among replicates was observed for direct PCR–P (18512.33, SD, 5553.77) in samples collected in February 2022 in Strožanac Beach, followed by B2 and B3 (mean 6274.33, SD 4093.18; mean 3650.33, SD 911.84, respectively). The average number of reads for K1 and K2 was 2523.67 (SD 215.26) and 2281.67 (SD 929.16) respectively. The lowest variability in the number of reads among samples was observed for K1. As reported for mock community samples, the lowest average coverage was observed for B1 (2202.67, SD 1794.00), where one sample failed the sequencing process with only 285 reads per sample (Supplementary Table 1).

As for samples collected in October 2020 in Kaštela Bay, the highest average number of reads with the highest variability among replicates was observed for B2 (57452.00, SD 20647.52), followed by P and B3 (mean 53064, SD 5193.10; mean 50457.50, SD 1298.96, respectively). The average coverage for B1 was 36855.00 (SD 19701.89), whilst the lowest average number of reads was observed for K1 (19769.33, SD 2374.20) (Supplementary Table 1).

#### 3.2.3. Bacterial community composition

At the ASV level, a total of 1742 ASVs were reconstructed for samples from 2020, of which 478 were retained after filtering and agglomerated to 139 taxa at the genus level. In 2022, a total of 650 ASVs were reconstructed and 123 retained after rare taxa removal, agglomerated to 62 genera. Raw ASV counts for marine samples collected in 2020 and 2022 are provided in Supplementary Data 4, 5 respectively, including taxonomy assignments and sample data information.

**FIGURE 5:**
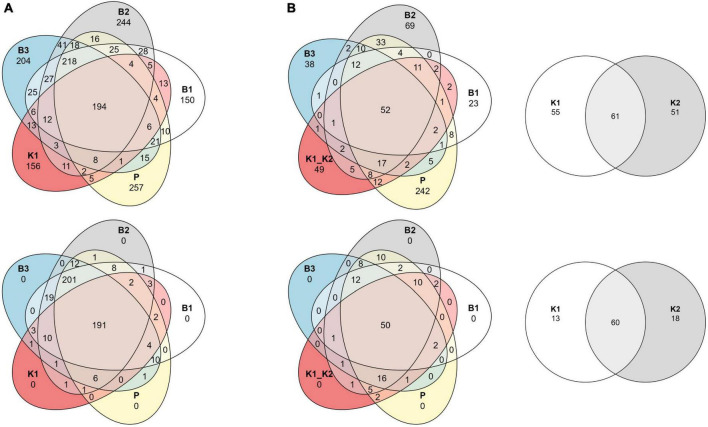
Venn diagrams of shared ASVs across DNA extraction methods as sample groups. Data are shown as an unfiltered dataset (upper) and filtered dataset for rare taxa (lower) for **(A)** 2020 and **(B)** 2022 marine samples collected in the eastern Adriatic Sea, respectively. B1: conventional phenol/chloroform/isoamyl extraction, B2 and B3: isopropanol and ethanol precipitation, respectively, K1: DNeasy PowerWater Kit (QIAGEN), K2: modified DNeasy PowerWater Kit (QIAGEN), P: direct PCR, Platinum Universal Master Mix (Invitrogen, Thermo Fisher Scientific).

Although sample read coverage and consequently number of reconstructed ASVs are subject to random sampling bias of the sequencing run, we report number of shared and distinct ASVs across methods to outline the effect of rare taxa filtering. Comparing the composition at the ASV level per method with the Venn diagram, the “core environmental microbiome,” i.e., ASVs detected by all methods, consisted of 194 and 191 ASVs, respectively, for unfiltered and filtered data for 2020, and 52 and 50 for 2022 (Figures 5A, B). Without the removal of rare ASVs from data analysis, each extraction method displayed a unique number of exclusive ASVs, which was not the case after filtering.

As for marine samples collected in 2020 in Kaštela Bay, the most relatively abundant phyla across all methods were *Proteobacteria* (mean 35.18%), *Bacteroidota* (mean 28.77%), and *Cyanobacteria* (mean 8.43%), except for K1 where *Actinobacteriota* showed the highest relative abundances (mean 33.36%). *Proteobacteria* were over-represented in P (mean 42.28%) whilst under-represented in K1 (mean 30.5%). *Cyanobacteria* were under-represented across K1 and P samples in respect to biochemically based methods B1, B2, and B3 (Supplementary Figure 1A). Interestingly, even at the kingdom level of taxonomic classification, besides Bacteria and Archaea, we noticed an ASV appearing across all methods (except in K1 samples) that was assigned as unclassified.

To our knowledge, this is the first report of marine bacterial community from Strožanac Beach in Croatia, although determining the microbial community structure of that site *per se* was not the main objective of this study. At the phyla level, in 2022 *Bacteroidota*, *Proteobacteria*, and *Cyanobacteria* were represented in highest relative proportions across all methods. *Bacteroidota* were the most abundant in P (mean 48.9%) and the least abundant in B1 (mean 37.08%) whilst on the contrary *Cyanobacteria* showed the highest relative abundances in B1 (mean 13.59%) and the lowest ones in P (mean 8.2%). *Proteobacteria* were the most abundant in P (mean 42.6%) and least abundant in B1 (mean 29.2%) (Supplementary Figure 1B).

The comparison of community structure between different DNA extraction methods and/or direct PCR amplification within each sampling time/site using PCA biplot on Aitchison distances of clr-transformed data on genus level is outlined in Figures 4C, D. Although there was a clear separation of kit based method and direct PCR from other methods in samples from 2020, the structure is not so clear in 2022 when the separation of direct PCR was retained, however, the samples extracted using kit-based methods showed greater variability as well as isopropanol and ethanol precipitations (B2 and B3). PERMANOVA again in both marine sample cases, flags these differences as statistically significant (*F* = 5.4558, *p* = 0.0002 for 2020; *F* = 1.6437, *p* = 0.0048 for 2022) (Table 2). The community structure was significantly different between direct PCR (P) and B1/B2/B3 and between kit-based methods and B1/B2/B3. This was reproduced for both sampling sites. Additionally, there is evidence that B1 separates significantly from B2/B3 methods for 2020, but not in 2022. However, due to the low number of unique permutations (35) in 2020 these differences need to be carefully considered (Table 2).

Finally, as by experimental design, all our tested communities should be different from one another (one mock, and two environmental samples taken at different sites and years, although still in Adriatic coastal area near Split). To glimpse at their mutual relationships, they were all visualized on the same PCA biplot (Supplementary Figure 2). Greatest source of variation was mutual separation of samples by origin/design that surpassed the one introduced by different methodological approaches. The only method that stood out slightly was K1 in 2020, but that was not reproduced in 2022.

## 4. Discussion

In this study, our main objective was to estimate the performances, strengths, and weaknesses of original phenol/chloroform/isoamyl DNA extraction protocol against its two shorter modifications were phenol step was omitted and DNA precipitated with isopropanol or ethanol, two kit-based protocols and a direct PCR approach circumventing DNA extraction step. We have compared DNA yields, NGS results and bacterial community composition between different methods on mock and environmental samples. The main goal was to estimate to what extent certain laborious/hazardous steps of classical phenol extraction might be replaced or other alternative procedures applied, especially for marine samples where the use of phenol is potentially unnecessary unless dealing with samples rich in organic content. Even though commercially available kits are replacing conventional phenol extractions nowadays, they are usually cost prohibitive and yield small amounts of DNA.

### 4.1. Mock community

As shown in previous research that evaluated different DNA extraction methods on both mock and environmental samples, mock community technical replicates in our study showed high within-method variability in DNA yields, however, the results were generally more in agreement for all methods for mock samples than environmental ones (Muñoz-Colmenero et al., 2021). The difference in achieved DNA yield was not statistically significant between extraction methods evaluated on PES filters for mock communities. The slightly lower yield achieved in our study with protocols based on the PowerWater Kit is in contrast with some of the previous findings where this protocol, which utilizes mechanical disruption of rigid bacterial cell walls, contributed to improved lysis efficiency, overall higher DNA yields, and is superior to extraction methods solely based on chemical lysis (Djurhuus et al., 2017). In accordance with previous research, purities of DNA extracts estimated *via* 260/280 and 260/230 absorbance ratios, which in our case were both higher and lower, respectively, than usually accepted for successful downstream analyses, did not predict unsuccessful PCR amplifications nor failed sequencing (Hart et al., 2015). Concerning NGS outputs, even though a slightly higher DNA yield was recovered with the conventional phenol/chloroform/isoamyl protocol and all PCR amplifications within our lab were successful, we have observed the tendency of these samples to result in lower read coverage. This could be a dire consequence of PCR inhibition for which residual traces of phenol are notoriously known for, even though amplicons in our lab had satisfactory purity values for downstream procedures of library preparation (Schrader et al., 2012). On the other hand, direct PCR generally lead to higher sample read coverage but also greater inconsistency between replicates, which could be linked to the fact that only miniature pieces of PES filters were utilized in the lysis protocol to acquire a DNA template for the amplification.

Similarly to other studies were sequencing outputs did not well represent true abundance of particular taxa, we have also observed departures in relative abundances of mock taxa from theoretically expected ones, regardless of the extraction method (Kembel et al., 2012; Gloor et al., 2017; Muñoz-Colmenero et al., 2021). Possible numerous contributors to this deviation could be attributed to usage of non-axenic cultures, kit residual bacterial contamination, sequencing capacity and accuracy, PCR primer bias, inaccurate taxonomic assignment, and/or variable 16S rRNA gene copy number (Caporaso et al., 2010; Pei et al., 2010; Kembel et al., 2012; Vetrovsky and Baldrian, 2013; Salter et al., 2014; Parada et al., 2016; Stoler and Nekrutenko, 2021). The patchiness that might have occurred during the preparation of mocks and the filtration process (due to possible adhesion to vessel surfaces, other cells or particles) might have also skewed the results (Djurhuus et al., 2017). However, all bacterial isolates and aliquots were thoroughly mixed before the filtration and all technical replicates within a method showed a similar trend in the bacterial community composition, therefore we propose that the mock community composition displayed here is a result of the (combined) biases mentioned above. Additionally, in an attempt to reduce the impact of PCR amplification on the error rates and chimeras formation, we used robust Q5 polymerase with the highest fidelity amplification available along with minimizing the number of PCR cycles when amplifying DNA extracts further to be sent to a sequencing platform (Sze and Schloss, 2019). Interestingly, a direct PCR protocol, which utilizes a different DNA polymerase, Platinum II Taq Hot-Start, together with a higher number of thirty-five cycles of amplification, generated similar results in terms of bacterial mock community composition as other DNA extraction protocols in combination with Q5 polymerase. The manufacturer (Invitrogen, Thermo Fisher Scientific, USA) claims this Platinum polymerase exhibits high sensitivity and specificity, inhibitors tolerance, universal primer annealing temperature, fast and robust amplification from AT- to GC-rich templates, and is compatible with Sanger sequencing. In our experience, we confirm the claims and report that all direct PCR amplifications were successful for the amplification of the V4-V5 region of the 16S rRNA without major prior optimization of direct PCR workflow.

Despite being added in similar proportions, the marine gram-positive *Microbacterium* sp. dominated in all mock communities’ samples across all methods evaluated in this study. The number of 16S rRNA gene copies reported for phylum Actinobacteria is 3.16 ± 1.7 per genome (Vetrovsky and Baldrian, 2013). However, copy numbers appear to be taxon-specific and, in some cases, even strain-specific. There are 28 *Microbacterium* sp. genomes reported to be in the range of 1 to 3 copies per genome according to the rrnDB database (Vetrovsky and Baldrian, 2013; Stoddard et al., 2015; Corretto et al., 2020). It is known that sequence variant reads are highly biased toward taxa with greater 16S gene copy numbers and that existing tools available for copy number predictions perform poorly for a vast majority of the genomes evaluated, thus emphasizing the remaining problem of gene copy number corrections which could potentially improve the relative quantitative estimates of microbial community compositions obtained *via* NGS (Kembel et al., 2012; Louca et al., 2018). On the other hand, it has been shown that 16S copy number may not be sufficient to describe bias in the PCR step (Brooks et al., 2015). For example, *S. agalactiae* had the largest copy number among the organisms in this study, but it was under-represented in respect to theoretical proportions expected for mock communities. In our experiment, interestingly, *Microbacterium* was the least over-represented across direct PCR samples, and we speculate that the reason for that could be the short lysis step before direct amplification which was not as efficient for the lysis of this Gram-positive bacteria’s thick peptidoglycan layer as in other methods tested.

Unlike the higher-than-expected relative abundances of *Microbacterium*, members of the marine *Flavobacteriaceae* family, which are crucial utilizers of various carbon polymers known to be numerically predominant in marine habitats (Gavriilidou et al., 2020), showed the lowest average relative abundances across all mock samples. *Vibrio*, *Pseudoalteromonas*, and *Glaciecola* were under-represented as well across all methods, yet, together with *Flavobacteriaceae*, showed slightly higher average relative abundances when isolated with the K2 protocol compared to other methods, indicating that these taxa, even though they are all Gram-negative bacteria, could be more prone to rigorous mechanical lysis than a purely chemical one. This could also be an indication that these taxa could potentially be under-represented in 16S metabarcoding studies of marine environments as well if the same extraction protocols, primer pair, and amplification conditions are applied. However, another additional important consideration that Hermans et al. (2018) emphasized and we would like to highlight is the awareness that the method’s ability to extract DNA from a particular taxon in mock communities does not necessarily correspond to the method’s ability to isolate DNA from the same taxon originating from far more complex environmental bacterial communities.

Compositional data analysis revealed that there were significant differences between DNA extraction based methods and direct PCR and are driven by both core mock and non-target taxa. Some of them are method specific and possibly related to prolonged (bio)chemical lysis of bacterial cells by some methods or lack of it in direct PCR. Generally, direct PCR showed smallest contribution of non-target taxa. Biochemically based methods B1, B2, and B3 show similar patterns and the isopropanol/ethanol precipitation method (B2 and B3, respectively) could substitute phenol-based methods as a shorter and considerably less hazardous alternative.

### 4.2. Environmental samples

Finding the appropriate DNA extraction protocol that results in a realistic representation of bacterial community for a given environment is crucial for DNA metabarcoding-based research. As previous studies have claimed, accurate identification of species present in a specific habitat and biodiversity estimates are far more critical than purely assessing quantities and qualities of DNA yielded with a particular extraction method (Deiner et al., 2015; Djurhuus et al., 2017; Hermans et al., 2018). In agreement with previous studies, we found significant differences in the composition of environmental bacterial communities investigated between different DNA extraction methods (Deiner et al., 2015; Djurhuus et al., 2017; Hermans et al., 2018; Liu et al., 2019; Muñoz-Colmenero et al., 2021). It seems there is not one superior extraction method among all that could be applied to every marine bacterial community, rather consistency in applying a certain technique of choice is essential to reduce biases inevitably introduced in the DNA isolation process and sample preparation. In agreement with Djurhuus et al. (2017), we have observed significant differences in DNA yields between different extraction protocols applied to marine samples. Overall significantly lower DNA yields were obtained with DNeasy PowerWater Kit than with other methods, as has been previously reported (Liu et al., 2019). However, a change in bead-beating technology QIAGEN introduced after 2020 resulted in superior DNA concentrations for 2022 marine samples that were more similar to other extraction methods. It has been revealed that DNA yields increase with the application of a smaller bead-beating system since the larger relative bacterial surface is occupied and greater numbers of bacteria could be more efficiently disrupted using smaller beads (Ma et al., 2020). Overall, in this study, the highest DNA yields were obtained with B1, B2, and B3 protocols based solely on the biochemical lysis and it seems that prolonged chemical lysis was efficient even for the disruption of marine Gram-positive bacteria, despite the frequently reported need for a bead-beating step (Kennedy et al., 2014; Deiner et al., 2015; Pollock et al., 2018). Although the protocols applied throughout the study were strictly followed, we observed that same methods sometimes behaved inconsistently in terms of yields obtained and extraction efficiency which might advocate for the application of more than one extraction method in one study on the same sample, if it is not cost-prohibitive. This might also aid in gaining more realistic insight into community structure. Especially sensitive to the choice of a particular extraction method are rare taxa, and the same conclusion was observed in our study where the use of different extraction methods influenced rarely observed ASVs the most. To improve comparability between methods, low-abundance OTUs, or in our case ASVs, are suggested to be removed (Liu et al., 2019).

Significant differences in community composition between different DNA extraction methods and direct PCR were observed for both marine samples, in 2020 and in 2022. This is in agreement with the striking differences between chemical and mechanical methods of lysis. Aggressive bead beating likely causes DNA shearing and fragmentation, possibly resulting in reduced detection of bacterial diversity while chemical lysis results in high-molecular-weight genomic DNA (Wintzingerode et al., 1997). Direct PCR circumvents these issues, however, it has resulted in different bacterial community composition driven by the detection of specific taxa, such as the Parvibaculaceae family or SAR86 clade, and reduced detection of Microbacterium or contaminants like Burkholderia-Caballeronia-Paraburkholderia. Both abundant as well as rare taxa are affected by the choice of DNA extraction method, however, rare taxa are even more so. In marine environmental samples B1, B2, and B3 performed with the least between-method differences, however, increased variability between replicates within-method was consistently reproduced between years. The main pitfall of our study is an uneven number of extraction replicates for the 2020 year which could be circumvented in further research with a higher and more consistent number of extraction technical replicates for each method and if possible, a greater sequencing depth. An internal DNA standard of a known quantity could be introduced in DNA extractions along with the sample and quantified by qPCR to estimate the extraction efficiency of each method, as was previously done (Boström et al., 2004).

## 5. Conclusion

Taking all into the account, we have found that there was not one superior DNA extraction method or experimental approach closest to the theoretically expected mock community composition, rather each displayed its advantages and disadvantages as well as particular preferences toward a specific taxon. This also generally applies to marine samples. There are many biases to be aware of introduced together with each DNA extraction technique, such as sampling strategies, storage conditions, 16S primer pair choice, PCR amplification conditions, library preparation, sequencing platforms, and bioinformatic analysis (Hermans et al., 2018; Pollock et al., 2018). Because significant preference toward a particular taxon is undeniably introduced, we suggest readers to make a cautious but most importantly persistent decision about the choice of the extraction method or direct PCR approach. From our data, we conclude that conventional phenol/chloroform protocol can be replaced with shorter and safer modifications thereof circumventing the phenol step, if purity of a starting material is not an issue. Concerning DNA extraction kits, since we have detected a few pitfalls with Dneasy PowerWater in combination with PES filters, other kits may be tested for different and/or similar sample matrices, such as DNeasy PowerSoil (QIAGEN), Fast DNA spin kit for soil (MP Biomedicals) or DNeasy Blood and Tissue Kit (QIAGEN) (Djurhuus et al., 2017; Walden et al., 2017; Liu et al., 2019). Direct PCR represents a possible approach when high throughput in sample processing is required, and it seems that lowered sample manipulation time translates to less non-target taxa detection.

## Data availability statement

The datasets presented in this study can be found in online repositories. The names of the repository/repositories and accession number(s) can be found in the article/Supplementary material.

## Author contributions

IS, IL, and ŽT designed the study. IS conducted the laboratory work with the contribution of ŽT, IL, and DŠ. IS and ŽT analyzed the data and preformed data visualization. IS drafted the manuscript. DŠ provided funding. All authors contributed to the article and approved the final submitted version.

## Footnotes

1
https://support.illumina.com/downloads/16s_metagenomic_sequencing_library_preparation.html
