# Supplementary material for: Evaluation of DNA extraction methods and direct PCR in metabarcoding of mock and marine bacterial communities
Source: Front Microbiol. 2023 Apr 17;14:1151907. doi: 10.3389/fmicb.2023.1151907 (PMC10149847; doi:10.3389/fmicb.2023.1151907)
Supplement: Supplementary file 1 [file Presentation_1.PDF]

Supplementary Material

Evaluation of DNA extraction methods and direct PCR in metabarcoding of mock and marine bacterial communities

Iva Stojan[1,2], Željka Trumbić[3*], Ivana Lepen Pleić[4] and Danijela Šantić[1]

[1] Laboratory of Marine Microbiology, Institute of Oceanography and Fisheries, Split, Croatia

[2] Doctoral Study of Biophysics, Faculty of Science, University of Split, Split, Croatia

[3] University Department of Marine Studies, University of Split, Split, Croatia

[4] Laboratory for Aquaculture, Institute of Oceanography and Fisheries, Split, Croatia

***Correspondence: Željka Trumbić**
e-mail: ztrumbic@unist.hr

Number of pages: 8

Number of Figures: 2

Number of Tables: 2

Number of Data: 5

1 Supplementary Figures and Tables

1.1 Supplementary Figures

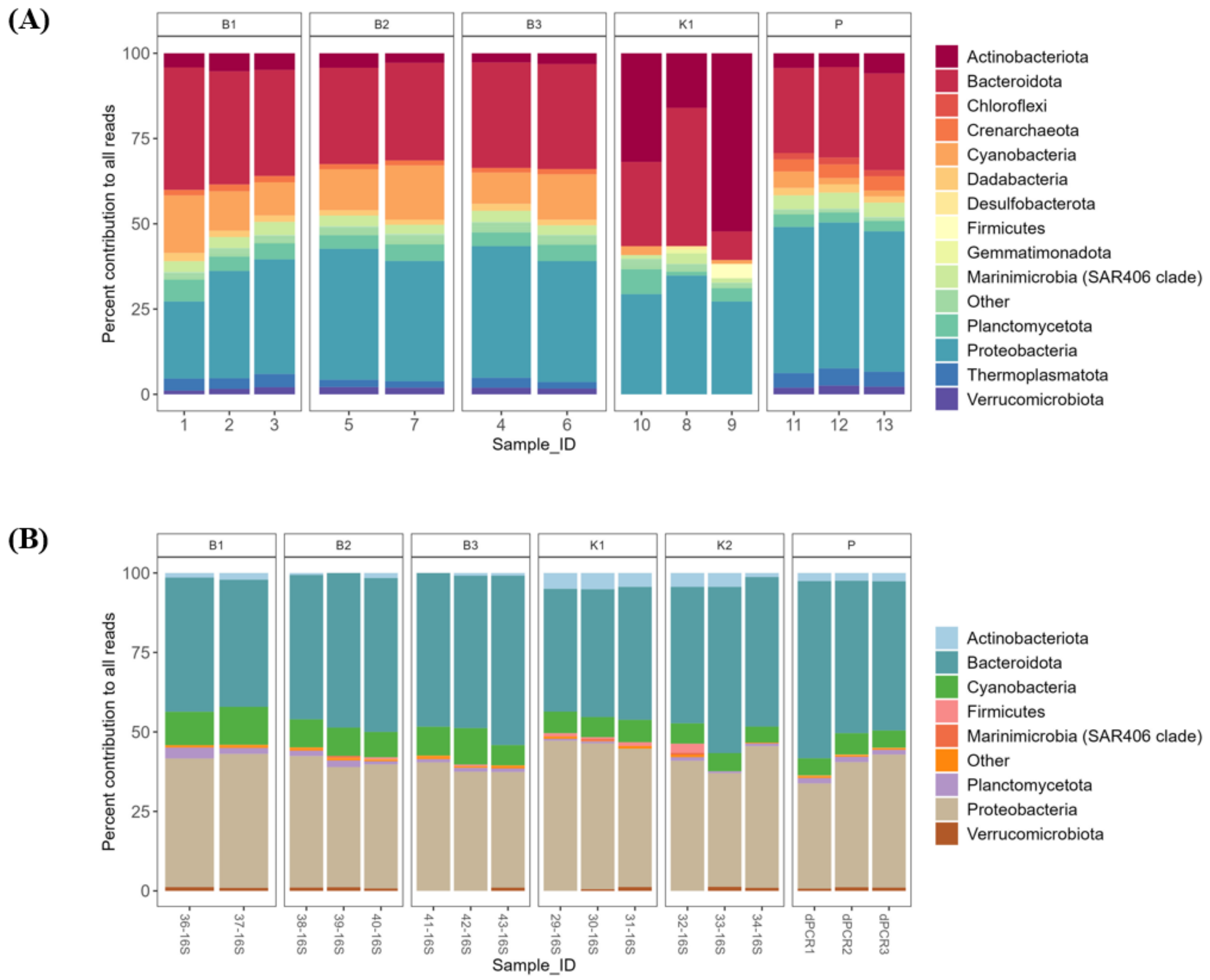

Supplementary Figure 1. Bar plots showing the taxa at phylum level detected from 0.22 µm PES filters from the marine environmental sample for 2020 (A) and 2022 (B) year, grouped by DNA extraction method. (B1: conventional phenol/chloroform/isoamyl extraction, B2 and B3: isopropanol and ethanol precipitation respectively, K1: DNeasy PowerWater Kit (QIAGEN), K2: modified DNeasy PowerWater Kit (QIAGEN), P: direct PCR, Platinum Universal Master Mix (Invitrogen, Thermo Fisher Scientific))

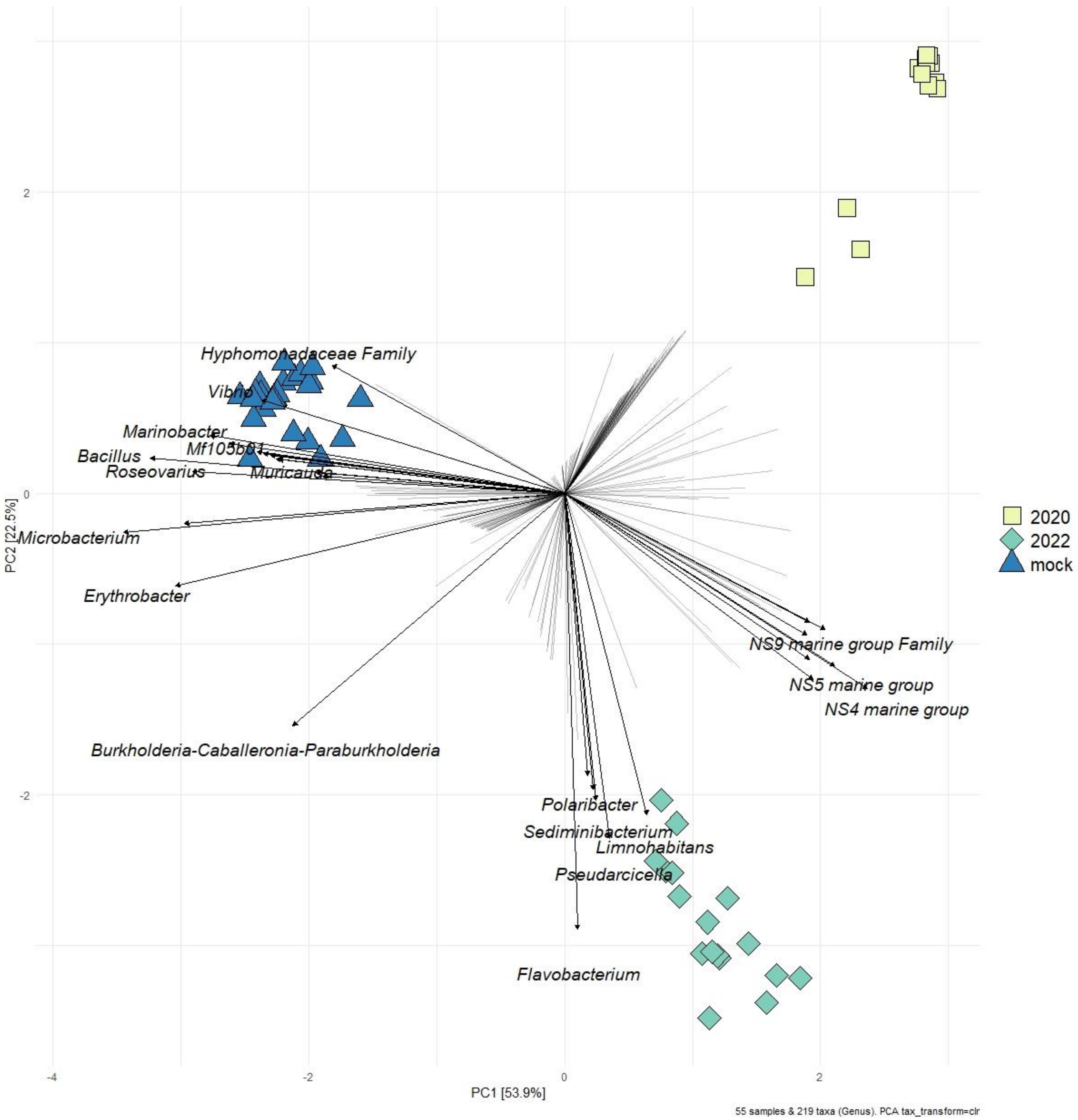

Supplementary Figure 2. Variance-based compositional principal component (PCA) biplot on Aitchison distances on clr-transformed ASV values with zero replacement using pseudo-counts, showing groupings of mock and environmental samples (2020 and 2022 year) with top taxa according to vector length agglomerated at the genus level.

1.2. Supplementary Tables

Supplementary Table 1: Number of reads per sample that made it through each step of dada2 pipeline; "input": number of raw reads, "filtered": filtered out low-quality sequences and tails, "denoisedF" and "denoisedR": denoised sequences after the core sample inference algorithm of forward and reverse reads respectively, "merged": merged sequences which are output if the forward and reverse reads overlap by at least 12 bases and are identical to each other in the overlap region, "nonchim": number of sequences after chimera removal. Samples 10-16S and 35-16S were discarded from further analyses due to unsatisfactory coverage. B1: conventional phenol/chloroform/isoamyl extraction, B2 and B3: isopropanol and ethanol precipitation respectively, K1: DNeasy PowerWater Kit (QIAGEN), K2: modified DNeasy PowerWater Kit (QIAGEN), P: direct PCR, Platinum Universal Master Mix (Invitrogen, Thermo Fisher Scientific)

Sample_ID	Method	input	filtered	denoisedF	denoisedR	merged	nonchim
MOCK COMMUNITY SAMPLES							
1-16S	K2	148050	73246	73071	73033	72544	71973
2-16S	K2	102885	66073	65891	65821	65441	64741
3-16S	K2	93632	57054	56832	56776	56185	55167
4-16S	K1	26067	14123	14066	13999	13774	13384
5-16S	K1	209148	142488	142126	142020	141190	138938
6-16S	K1	162192	106225	105950	105968	105279	103611
8-16S	B1	12004	8281	8206	8174	7983	7772
9-16S	B1	7212	4712	4655	4531	4366	4265
10-16S	B1	348	201	180	174	169	167
11-16S	B1	3032	1825	1797	1783	1738	1735
23-16S	B1	2723	1419	1351	1335	1276	1273
12-16S	B2	16038	8899	8830	8779	8627	8574
13-16S	B2	58830	40477	40274	40128	39653	39043
14-16S	B2	28062	18255	18121	18109	17858	17674
15-16S	B2	6862	4634	4576	4541	4399	4356
24-16S	B2	98342	53325	53097	53041	52362	51578
25-16S	B2	54600	39116	38880	38797	38343	37895
16-16S	B3	10238	6977	6897	6820	6604	6563
17-16S	B3	219478	110818	110522	110524	109219	106028
18-16S	B3	214378	128246	127821	127715	126060	121166
19-16S	B3	17950	9598	9515	9465	9133	8977
26-16S	B3	9046	6873	6727	6689	6455	6315
27-16S	B3	24477	18036	17804	17751	17348	16987
dPCR4	P	73721	61108	60937	60897	60490	59263
dPCR5	P	436222	355294	353623	354203	349089	334541

dPCR6	P	21484	17856	17718	17603	17097	16177
MARINE SAMPLES 2022							
29-16S	K1	2603	2273	2036	1971	1603	1561
30-16S	K1	2688	2259	2041	2046	1650	1578
31-16S	K1	2280	1937	1763	1722	1394	1310
32-16S	K2	1553	1319	1162	1094	908	857
33-16S	K2	1964	1655	1474	1462	1159	1142
34-16S	K2	3328	2922	2685	2594	2117	2037
35-16S	B1	285	219	157	132	112	108
36-16S	B1	2483	1975	1782	1739	1427	1395
37-16S	B1	3840	3287	3031	2979	2482	2400
38-16S	B2	5161	4158	3856	3798	3220	3148
39-16S	B2	2853	2198	1973	1971	1686	1630
40-16S	B2	10809	8701	8217	8182	7124	6965
41-16S	B3	3446	2720	2527	2369	1948	1875
42-16S	B3	2858	2349	2114	2070	1673	1609
43-16S	B3	4647	3786	3484	3359	2726	2659
dPCR1	P	15592	14326	13539	13353	11281	10751
dPCR2	P	15028	13704	12987	12680	10680	10190
dPCR3	P	24917	22848	21494	21284	17682	16654
MARINE SAMPLES 2020							
1	B1	28783	25250	24681	24632	23298	22832
2	B1	22471	19704	19223	19186	18173	17896
3	B1	59311	51895	51193	51086	49492	48862
4	B3	49539	43104	42220	42221	40269	39727
5	B2	72052	62147	61286	61331	59507	58656
6	B3	51376	45118	44385	44171	42676	42005
7	B2	42852	37608	36902	36862	35448	34915
8	K1	21115	17932	17800	17729	17375	16999
9	K1	21165	18279	18169	18218	17966	17710
10	K1	17028	14897	14683	14694	14363	14049
11	P	47636	42218	41026	40826	37874	36964
12	P	53571	47185	46137	45830	42724	41511
13	P	57985	49356	48196	47947	44610	43218

Supplementary Table 2. DNA concentrations (ng/µL) with average values and standard deviations per extraction method and absorbance ratios (A260/280 and A260/230) given for each sample. B1: conventional phenol/chloroform/isoamyl extraction, B2 and B3: isopropanol and ethanol precipitation respectively, K1: DNeasy PowerWater Kit (QIAGEN), K2: modified DNeasy PowerWater Kit (QIAGEN). The elution volume was 35 µL.

Sample_ID	Extraction method	DNA concentration (ng/µL)	Average DNA concentration per method (ng/µL)	Standard deviation for DNA concentrations	A260/280	A260/230
			MOCK			
1-16S	K2	3.95	4.85	1.56	2.1	0.121
2-16S	K2	6.65			3.4	0.05
3-16S	K2	3.95			2.3	0.13
4-16S	K1	3.85	5.52	1.44	2.3	0.21
5-16S	K1	6.4			2.6	0.07
6-16S	K1	6.3			2.571	0.062
8-16S	B1	7.2	6.44	1.00	4.1	0.08
9-16S	B1	6.85			3.4	0.11
10-16S	B1	6.65			3.8	0.1
11-16S	B1	6.8			2.9	0.11
23-16S	B1	4.678			3.13	0.17
12-16S	B2	6.9	5.89	1.76	3.26	0.08
13-16S	B2	7.5			2.52	0.127
14-16S	B2	5.5			1.9	0.144
15-16S	B2	7.7			3.6	0.44
24-16S	B2	4.146			3.06	0.172
25-16S	B2	3.573			3.9	0.104
16-16S	B3	7.95	6.12	1.74	3.7	0.096
17-16S	B3	7.35			3.1	0.23
18-16S	B3	6.55			2.65	0.18
19-16S	B3	5.5			3.01	0.47
26-16S	B3	6.34			2.91	0.26
27-16S	B3	3			2.71	0.31
			MARINE SAMPLES 2022			
29-16S	K1	6.8	10.22	2.96	1.789	0.925
30-16S	K1	12.05			2.042	0.115
31-16S	K1	11.8			1.86	0.268
32-16S	K2	6.9	8.20	2.17	1.76	0.715
33-16S	K2	7			1.8	0.296
34-16S	K2	10.7			1.981	0.169

35-16S	B1	23.95	28.78	4.68	1.988	2.325
36-16S	B1	29.1			1.914	1.83
37-16S	B1	33.3			1.947	1.903
38-16S	B2	23.2	16.95	6.04	1.617	1.805
39-16S	B2	16.5			1.634	1.976
40-16S	B2	11.15			1.828	0.746
41-16S	B3	11.642	11.96	1.50	1.642	1.705
42-16S	B3	13.6			1.732	1.609
43-16S	B3	10.65			1.7	1.127
MARINE SAMPLES 2020						
1	B1	13.300	29.12	16.38	1.834	2.031
2	B1	46.000			2.135	2.7
3	B1	28.050			1.962	2.892
4	B3	51.05	58.20	10.11	2	2
5	B2	121.5	129.63	11.49	2	2.6
6	B3	65.35			2	1.9
7	B2	137.75			2	2.3
8	K1	5.85	3.38	2.14	3.9	0.047
9	K1	2.1			1.7	0.3
10	K1	2.2			1.9	0.173

2. Supplementary Data

Supplementary Data 1: Detailed protocols of isopropanol (B2) and ethanol (B3) precipitation for DNA extraction from 0.22 µm PES filters

Supplementary Data 2: Relative abundances (%) of mock constituents (filtered mock dataset) agglomerated at the family level, shown per method: B1- conventional phenol/chloroform/isoamyl extraction, B2 and B3- isopropanol and ethanol precipitation respectively, K1- DNeasy PowerWater Kit (QIAGEN), K2- modified DNeasy PowerWater Kit (QIAGEN), P- direct PCR, Platinum Universal Master Mix (Invitrogen, Thermo Fisher Scientific)

Supplementary Data 3: ASV table, taxa table and sample data for mock community samples. B1- conventional phenol/chloroform/isoamyl extraction, B2 and B3- isopropanol and ethanol precipitation respectively, K1- DNeasy PowerWater Kit (QIAGEN), K2- modified DNeasy PowerWater Kit (QIAGEN), P- direct PCR, Platinum Universal Master Mix (Invitrogen, Thermo Fisher Scientific)

Supplementary Data 4: ASV table, taxa table and sample data for marine samples from the 2020 year. B1- conventional phenol/chloroform/isoamyl extraction, B2 and B3- isopropanol and ethanol precipitation respectively, K1- DNeasy PowerWater Kit (QIAGEN), K2- modified DNeasy PowerWater Kit (QIAGEN), P- direct PCR, Platinum Universal Master Mix (Invitrogen, Thermo Fisher Scientific)

Supplementary Data 5: ASV table, taxa table and sample data for marine samples from the 2022 year. B1- conventional phenol/chloroform/isoamyl extraction, B2 and B3- isopropanol and ethanol precipitation respectively, K1- DNeasy PowerWater Kit (QIAGEN), K2- modified DNeasy PowerWater Kit (QIAGEN), P- direct PCR, Platinum Universal Master Mix (Invitrogen, Thermo Fisher Scientific)
