# Supplementary material for: Evaluation of DNA extraction methods and direct PCR in metabarcoding of mock and marine bacterial communities
Source: Front Microbiol. 2023 Apr 17;14:1151907. doi: 10.3389/fmicb.2023.1151907 (PMC10149847; doi:10.3389/fmicb.2023.1151907)
Supplement: Supplementary file 2 [file Data_Sheet_1.DOCX]

**Supplementary Data 1: Isopropanol and ethanol precipitation protocols for DNA extraction from** **0.22 μm PES filters (detailed protocols B2 and B3)**

1. Cut the 0.22 μm PES filter (diameter 47 mm) with a clean scalpel into smaller pieces.
2. Add 750 µL of 10x TEN lysis buffer (prepare fresh: 0.1 M Tris-HCl, 0.01 M EDTA pH 8.0, 1 M NaCl, pH 8.0) to each tube and vortex thoroughly.
3. Incubate for 1 hour at room temperature.
4. Add 25 μl of TEN buffer supplemented with 1mg/ml lysozyme (freshly prepared) to each sample. Mix thoroughly by inverting. From this point do not vortex.
5. Incubate for 1.30 h at 37°C in the heating block.
6. Add 0.2 mg/ml of Proteinase K to each sample.
7. Add 40 μl 20% SDS and gently resuspend.
8. Incubate for 1 hour at 65°C, followed by 10 min at 95°C (heating block).
9. Centrifuge at 18000 RCF for 20 min at room temperature.
10. Transfer the aqueous layer (~700µL), avoiding cell debris, into clean 2 ml tube.
11. For isopropanol precipitation: add one volume of isopropanol (protocol B2); for ethanol precipitation: add two volumes of absolute ethanol (protocol B3).
12. Incubate overnight at –20°C.
13. Centrifuge at 20000 RCF for 30 min at 4°C.
14. Discard the supernatant and wash the pellet twice with 500 µl cold 70% ethanol.
15. Centrifuge at 20000 RCF for 10 min at 4°C.
16. Discard and remove all remaining ethanol (centrifuge shortly and pipet).
17. Air dry the pellet or shortly incubate at 37°C until all the ethanol evaporates.
18. Resuspend pellet in 35 μl clean H_2_O or 1x TE buffer (10 mM Tris, 1 mM EDTA, pH ~8.0).
